# Proteome analysis of the *Mycobacterium tuberculosis* Beijing B0/W148 cluster

**DOI:** 10.1038/srep28985

**Published:** 2016-06-30

**Authors:** Julia Bespyatykh, Egor Shitikov, Ivan Butenko, Ilya Altukhov, Dmitry Alexeev, Igor Mokrousov, Marine Dogonadze, Viacheslav Zhuravlev, Peter Yablonsky, Elena Ilina, Vadim Govorun

**Affiliations:** 1 Federal Research and Clinical Centre of Physical-Chemical Medicine, Moscow, Russian Federation; 2 Moscow Institute of Physics and Technology, Dolgoprudny, Russia; 3 St. Petersburg Pasteur Institute, St. Petersburg, Russian Federation; 4 Research Institute of Phtisiopulmonology, St. Petersburg, Russian Federation

## Abstract

Beijing B0/W148, a “successful” clone of *Mycobacterium tuberculosis*, is widespread in the Russian Federation and some countries of the former Soviet Union. Here, we used label-free gel-LC-MS/MS shotgun proteomics to discover features of Beijing B0/W148 strains that could explain their success. Qualitative and quantitative proteome analyses of Beijing B0/W148 strains allowed us to identify 1,868 proteins, including 266 that were differentially abundant compared with the control strain H37Rv. To predict the biological effects of the observed differences in protein abundances, we performed Gene Ontology analysis together with analysis of protein-DNA interactions using a gene regulatory network. Our results demonstrate that Beijing B0/W148 strains have increased levels of enzymes responsible for long-chain fatty acid biosynthesis, along with a coincident decrease in the abundance of proteins responsible for their degradation. Together with high levels of HsaA (Rv3570c) protein, involved in steroid degradation, these findings provide a possible explanation for the increased transmissibility of Beijing B0/W148 strains and their survival in host macrophages. Among other, we confirmed a very low level of the SseA (Rv3283) protein in Beijing B0/W148 characteristic for all «modern» Beijing strains, which could lead to increased DNA oxidative damage, accumulation of mutations, and potentially facilitate the development of drug resistance.

*Mycobacterium tuberculosis* (MTB) is the causative agent of tuberculosis (TB) and, according to the Global Tuberculosis Report produced by the World Health Organization (WHO), nine million people had TB in 2014 and 1.5 million died because of the disease^1^. Of note, 80% of TB cases are concentrated in 22 “high-burden” countries. The Russian Federation belongs to this list and has a relatively high rate of new TB cases (80/100,000 population/year) according to WHO statistics^2^. Analysis of the MTB population structure in The Russian Federation has defined three main genetic families, Ural, LAM and Beijing^3^. According to earlier studies, more than 50% of all MTB strains isolated in Russia belong to the Beijing family, and a quarter of them are the Beijing B0/W148 variant^4^,^5^.

A recent systematic and critical review summarized various biological and phylogenetic features of the Beijing B0/W148 cluster^6^. Strains of this cluster possess unique pathogenic properties, including stronger association with multidrug resistance and higher levels of clustering (i.e. higher transmissibility) compared with other Beijing variants, as demonstrated by a meta-analysis of studies from across the former Soviet Union^6^. Additionally, members of this cluster demonstrate increased virulence in a macrophage model^7^, although in a mouse model, no increased virulence was observed^8^. Beijing variant MTB strains have a unique genome organization; recently, we reported large scale chromosomal inversions spanning 350 and 550 kb segments of the chromosome^9^. The presence of these inversions in Beijing B0/W148 cluster strains was confirmed by PCR, sequencing, and RFLP analysis. In addition, we identified Beijing B0/W148 cluster-specific SNPs. However, the inversions and the SNPs are insufficient to explain the success of the Beijing B0/W148 cluster. Hence, there is a particular interest in studying the proteomes of these pathogens, which will extend the genomic data by allowing detailed analyses of protein abundance, as well as protein-protein interactions.

According to the TubercuList database, a total of 4,018 proteins are encoded in the genome of *M. tuberculosis* H37Rv strains^10^. Not long ago the majority of MTB proteomic studies focused on the analysis of protein groups and individual proteins involved in certain processes, for example, in the development of drug resistance^11^,^12^,^13^,^14^. The constant technological improvements in analysis methods for biomolecules have made it possible to apply discovery driven shotgun proteomics approaches to the investigation of MTB, with a focus on the identification and quantification of the whole proteome of these strains. The most comprehensive proteome of *M. tuberculosis* Н37Rv was described recently by Schubert *et al*.^15^. The authors used discovery-driven mass spectrometry analysis based on extensive off-gel fractionation followed by LC-MS/MS to identify and quantify 3,074 proteins, whereas the implementation of gel-LC-MS/MS for shotgun proteomics allows the identification of about 2,000 proteins^16^,^17^. However, relatively few studies have focused on the proteomes of specific genetic families of MTB and only two reports characterizing the proteomes of Beijing family strains have been published. De Souza *et al*.^18^ described the proteomic profiles of hypo- and hypervirulent clinical Beijing isolates, whereas de Keijzer *et al*. disclosed the proteomic features of MTB strains belonging to ancient (atypical) and modern (typical) sublineages of the Beijing family^19^.

In this study, we have applied a label-free gel-LC-MS/MS shotgun proteomics approach for empirical ‘bottom-up’ exploration of Beijing B0/W148 strains.

## Results

### Selection of *M. tuberculosis* strains for proteome analysis

Seven Beijing B0/W148 cluster strains were selected for inclusion in the proteomic study. Whole genome sequencing of five of the seven strains had been performed previously (Table 1). All studied Beijing strains carried the large scale chromosomal inversions, spanning 350 and 550 kb segments of the chromosome, which we described previously^9^. The laboratory H37Rv strain was used for comparative analysis. Each strain was grown in three biological replicates, independently, to give a total of 24 samples. Bacterial cells were collected in stationary phase, and total proteins were extracted.

### Comprehensive proteome analysis of *M. tuberculosis*

For comprehensive proteomic analysis via LC-MS/MS, the proteins from the seven Beijing B0/W148 cluster strains and H37Rv were fractionated by SDS-PAGE, followed by in-gel tryptic digestion and analysis of the resulting peptide mixtures. The combined analysis yielded a total of 1,098,994 MS/MS spectra, of which 366,621 were assigned to unique peptide sequences using two different MS/MS search algorithms (peptide FDR < 1%).

For the H37Rv strain we identified a total of 1,560 proteins with a minimum of two unique peptides in two biological replicates. For the seven Beijing B0/W148 samples 1,868 proteins were identified, of which 1,176 (>60%) were identified in all strains. Identified proteins and peptides are presented in Tables S1–S3.

**Figure 1:**
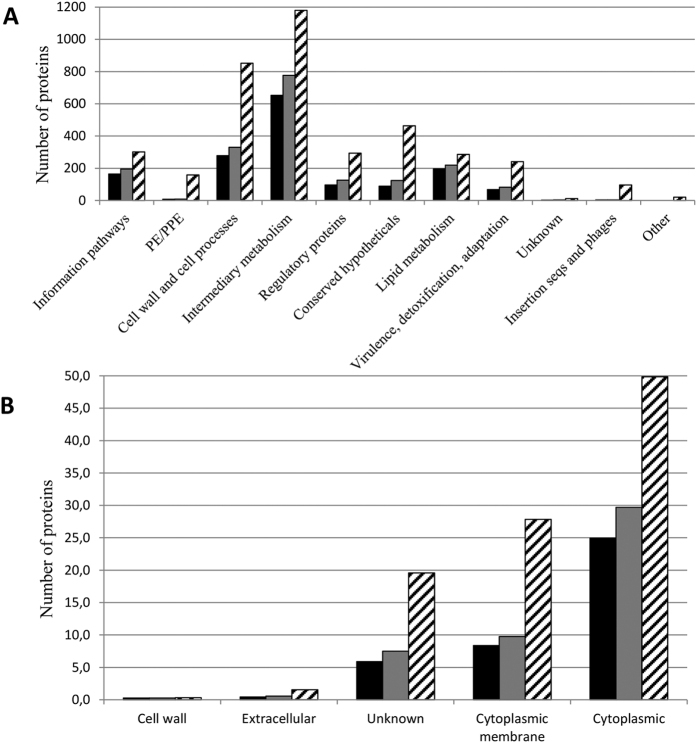
Functional distribution of the proteins identified by LC-MS/MS. Proteins present in our MS dataset for H37Rv (black bars) and Beijing B0/W148 (gray bars) and all annotated genes (black/white-banded bars) were categorized by. (**A**) Functional class categories according to TubercuList v 2.6 (http://tuberculist.epfl.ch/). (**B**) Localization as given by PSORTdb v 3.0 (http://db.psort.org/).

We compared the numbers of identified proteins in H37Rv and the Beijing strains in different functional categories (as defined by TubercuList) and subcellular localizations (as defined by PSORTdb) and did not find any biases between H37Rv and the Beijing B0/W148 strains (Fig. 1A,B).

### Qualitative proteome analysis of *M. tuberculosis* strains

Initially, qualitative proteome analysis was performed to compare proteins identified in the group of Beijing B0/W148 cluster strains with those from H37Rv. To achieve this, we created two lists of proteins; the first included proteins identified in five of seven Beijing B0/W148 cluster strains and the second comprised proteins identified both from H37Rv in our study and in the study of Schubert *et al*.^15^.

In this way, we identified 17 proteins characteristic of the Beijing B0/W148 strains that were not detectable in H37Rv. The majority of these were also identified in Beijing strains in a recent report^18^. In addition, 57 proteins not detectable from the Beijing B0/W148 strains were present in H37Rv (Table S4).

The available WGS dataset for five of the Beijing B0/W148 strains was used to estimate the concordance between genomic and proteomic data. We found genetic changes with potential to explain the presence of 8 of the 17 (47%) of Beijing B0/W148 specific proteins. In H37Rv the upstream region of the Rv2277c, Rv2475c, and Rv3323c genes carries the IS*6110* element, which is missing in the Beijing B0/W148 strains and is likely to affect gene expression. In addition, there are three CG repeats present in the Rv2974c upstream region in the H37Rv genome, while the Beijing B0/W148 genomes contain two such repeats. We also detected a single nucleotide insertion in the Rv0976c upstream region and non-synonymous SNPs (nsSNPs) in the Rv0945, Rv1319c, and Rv2351c coding regions of Beijing B0/W148 strains, relative to that of H37Rv.

Among the 57 proteins that were not detectable in the proteomes of Beijing B0/W148 strains but present in the proteome of H37Rv, 33 carried genetic mutations compared to the H37Rv genome. Among these, six genes (Rv0072 (part of RD105), Rv1576c and Rv1586c (part of RD149), Rv1762c (part of RD152), Rv2263 (part of RD181) and Rv2818c (part of RD207)) mapped to chromosome regions showing differences between the two groups of strains^20^. The absence of two proteins from the Beijing B0/W148 group can be explained by an insertion (Rv0888: 987586 insGG) and a deletion (Rv1997: 2241032 delG) in the coding regions of their respective genes, which both lead to sequence changes causing protein coding frameshifts. In addition, we found changes in the upstream regions of three genes and a further 22 genes carried nsSNPs in their coding regions (Table S4).

### Quantitative proteome analysis of *M. tuberculosis* strains in the Beijing B0/W148 cluster

The abundance of proteins in Beijing B0/W148 cluster strains was compared to that in H37Rv using Progenesis LC-MS software. For this experiment, we limited our analysis to the 1,016 proteins identified in both experimental groups (Tables S5 and S6; Figure S1). In total, we identified 192 proteins with abundances that were significantly different between the two groups (p < 0.05). Among these, 24 were over-represented in the Beijing B0/W148 cluster strains and 168 under-represented (Table S4). Worth noting, we considered all significant alterations in protein abundance, without thresholds for fold change, to allow for maximum identification of differentially abundant proteins.

Genes encoding proteins with significant differences in levels of abundance (n = 192) were matched to known operons of the H37Rv genome using MicrobesOnline Operon Predictions (www.microbesonline.org)^21^, resulting in the identification of 30 genes in 11 operons. In the majority of cases we observed changes in protein abundances in only one direction (over or under) for genes encoded by the same operon. However, changes in two genes in the same operon, Rv1380 (over) and Rv1384 (under), resulted in opposite changes in the abundances of the corresponding proteins.

We also found insertions (Rv3234c: 3610391 insC), deletions (Rv0927c: 1034211 delTGC; Rv1884: 2094915 delCGTCAG) and 35 nsSNPs in the coding regions of genes encoding proteins with different abundances in the two strains. Additionally, we searched for mutations in transcription initiation sites and transcription factor (TF) binding sites within our genomic data^22^,^23^. Mutations in TF binding sites for Rv0169, Rv1129c, Rv1872c and in transcription start sites for Rv0169, Rv1196, Rv1508c, Rv1872c, Rv2627c, Rv2711 were identified (Table S4).

### Functional characteristics of differentially abundant proteins

To determine possible cumulative effects of the differentially abundant proteins on the function of mycobacterial cells, we combined the results of qualitative and quantitative proteome analysis. Thus, proteins exclusively identified in or not detectable from the Beijing B0/W148 cluster strains were attributed to the groups of over- or under-represented proteins, respectively; the extended over- and under-represented groups consisted of 41 proteins and 225 proteins, respectively (Table S4, Figure S1).

Proteins were classified according to the Gene Ontology (GO) annotations “biological process” (BP), “cellular component” (CC) and “molecular function” (MF) using the PANTHER classification system (Figure S2A). In case of “molecular function” term both under- and over-represented proteins enriched “oxidoreductase activity” and “transferase activity” sub-categories of GO “catalytic activity” and additionally “transporter activity” category. For “biological process” term over-represented proteins enriched “primary lipid metabolic process”, whereas under-represented proteins were distributed across different categories (Table 2, Figure S2).

**Figure 2:**
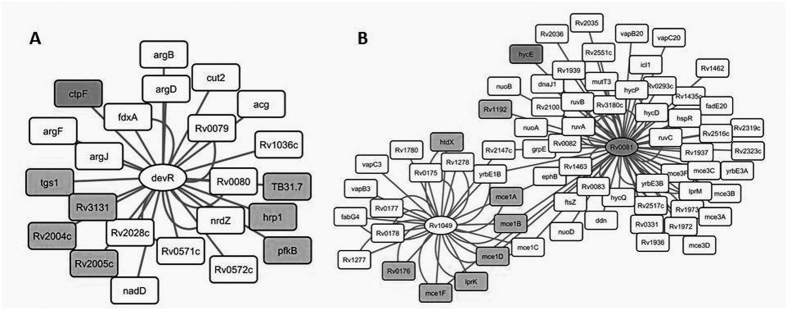
Network map of co-regulated groups of genes. Ellipse and rectangles indicates TF and genes, respectively. Gray color indicates genes which correspond to under-represented proteins. White color indicates genes which correspond to proteins without changes or unidentified proteins. (**A**) Group of genes bound by TF devR/DosR; (**B**) Group of genes bound by TFs Rv0081 and Rv1049.

Given the regulon organization of prokaryotic genes and operons, we hypothesized that proteins controlled by a single transcription factor must be assembled into “unidirectional change” groups. To verify this hypothesis we analyzed 266 differentially abundant proteins by mapping them to a gene regulatory network consisting of 65 TFs and 431 genes regulated by these TFs^24^. Visualization by Cytoscape v 2.8.3 software allowed us to position the proteins in the network. Five and 38 proteins from the over- and under-represented groups, respectively were found to belong to this gene network (Table 3, Fig. 2).

Next, we focused our attention on co-regulated groups of proteins controlled by the same TF and identified a set of five TFs responsible for the regulation of 24 genes. Of these TFs, Rv3133c, Rv1049 and Rv0081 had the most extensive connections and Rv3133c, a key member of the DosR regulon, was associated with eight under-represented proteins, Rv1997, Rv2004c, Rv2005c, Rv2029c, Rv2623, Rv2626c, Rv3130c and Rv3131 (Fig. 2A). Therefore we examined the abundance profiles of proteins belonging to the DosR regulon, which consists of 52 genes and the sensor histidine kinase, dosT^15^. In this study, we identified 33 of the 53 DosR proteins (62%), 11 of which were under-represented in strains of the Beijing B0/W148 cluster (Figure S1). Most of the 11 under-represented proteins have been proposed to be involved in lipid transport and degradation and are likely to function in the assimilation of exogenous lipids from host cell membranes. Interestingly, no difference in the abundance of DosR transcription factor itself was observed between Beijing B0/W148 strains and H37Rv.

Another group included the TFs Rv1049 and Rv0081, which regulate the synthesis of eight proteins under-represented in Beijing B0/W148 strains (Fig. 2B). Of these eight, six proteins, Rv0169, Rv0170, Rv0172, Rv0173, Rv0174, and Rv0176, are involved in membrane transport of phospholipids and belong to the ABC transporter family. Of note, Rv0081, which was recently proposed to be a hypoxia regulator^25^,^26^, is a member of the ArsR/SmtB family of metal-dependent transcriptional regulators and is directly regulated by the response regulators DosR/DevR and MprAB^27^. Our results indicate that MprA (Rv0981) is under-represented in Beijing B0/W148 cluster strains.

## Discussion

According to previous studies, the Beijing genotype represents approximately 50% of MTB strains in East Asia and at least 13% of strains worldwide^28^. Among them the Beijing B0/W148 clonal cluster, can be distinguished. It is defined as a “successful” Russian clone of MTB^6^ and is known under different names, initially as B0 or W148^5^,^29^, or, more recently, as clade B of the “East European” sublineage^3^, or the Resistant European Tuberculosis cluster^30^. The pathobiology, genomic characteristics and phylogeography of the B0/W148 clonal cluster are described in a recent systematic and critical review^6^, although many questions concerned its pathogenomics remain unclear.

In this study we focused on the specific features of the proteome of the Beijing B0/W148 clonal cluster strains. For this reason, we selected seven МТВ strains of this cluster with a similar VNTR-profile and containing the specific chromosomal rearrangements. The well-studied laboratory H37Rv strain was used for comparison.

For the proteomic analysis we used discovery-driven MS, also known as the shotgun MS approach, aimed at maximizing proteome coverage. The first studies using this method to investigate MTB were published in 2004^31^ and at the time of writing there were 30 such articles in the PubMed database, including two ones focused on the Beijing family. In 2010 Souza *et al*. applied a label-free gel-LC-MS/MS to identify 1,668 proteins in hyper- and hypovirulent MTB Beijing isolates^18^. The other study of the Beijing MTB family derived 2,392 proteins using a label-based SCX-LC-MS/MS approach, and described the differences between ancient and modern Beijing strains^19^. In our study we identified 1,951 proteins for Beijing B0/W148 strains and 1,560 ones for H37Rv, which is comparable to the number of proteins identified in earlier reports.

It is known that different MTB genetic groups can exhibit different features that affect protein extraction. Because we used the same workflow both for Beijing B0/W148 and H37Rv strains, to be sure of its effectiveness we compared the number of identified proteins within functional categories and subcellular localizations for tested groups (Fig. 1A,B). Both groups showed a comparable distribution of proteins across functional categories, in agreement with the results of de Keijzer *et al*.^19^, and subcellular localizations. It allows us to conclude that the differences in protein abundance we observed between Beijing B0/W148 and H37Rv strains are independent from our workflow and reflect the true physiological characteristics of pathogens.

For a detailed description of the specific properties of the Beijing B0/W148 cluster, we performed a comparative proteomic analysis of these strains with H37Rv using a combination of qualitative and quantitative proteomic data. In total, 266 differentially abundant proteins were identified, of which 41 were over- and 225 were under-represented, respectively, in Beijing B0/W148 cluster strains.

By analyzing the sequences of available Beijing B0/W148 genomes, we found possible explanations for 47% of the changes revealed by qualitative proteomic analysis. Specifically, we demonstrated that the absence of six proteins was due to the fact they mapped to five deleted regions, a characteristic feature of the whole Beijing family^32^. Additionally, we identified nsSNPs in coding regions and nucleotide substitutions/IS*6110* insertions in the upstream regions of genes encoding 33 proteins that were present or not detectable in the Beijing B0/W148 cluster strains. However, the majority of differentially abundant proteins identified by quantitative proteomic analysis could not be explained by differences in the genomic data.

Two complementary approaches were used to predict the functional effects of the observed features of the Beijing B0/W148 proteome. We first applied GO analysis to identify functional categories enriched for the differentially abundant proteins (n = 266). We believe that the distribution of proteins categorized by the GO annotation “biological processes” is most relevant. Accordingly, we found that a substantial group of differentially abundant proteins (33/266, 12.4%) belong to the “metabolic process” category (GO:008152). In-depth analysis revealed an enrichment for over-represented proteins (4/41, 9.8%) in the GO:0006629 category, “lipid metabolic process”, while under-represented proteins were relatively equally distributed among the categories: “lipid” (GO:0006629), “cellular amino acid” (GO:0006520) and “carbohydrate” (GO:0005975) metabolic processes.

Among the over-represented proteins involved in lipid biosynthesis, the long-chain-fatty-acid-CoA ligase, FadD15, is known to be the one of seven fatty-acid-CoA synthases induced in virulent strains^33^. Another protein, AgpS, is an alkyl-DHAP synthase that initiates lipid anabolism. Together, these results suggest lipid synthesis is upregulated in Beijing B0/W148 cluster strains. Consistent with this hypothesis, the majority of proteins involved in fatty acid catabolism were under-represented in Beijing B0/W148 cluster strains (Table 2). By contrast, the HsaA oxygenase subunit of the flavin-dependent monooxygenase, encoded by one of 126 genes necessary for survival in macrophages^34^, was over-represented in Beijing B0/W148 cluster strains. This protein is involved in the catabolism of steroids and could have important effects on the infected host by reducing the local concentration of membrane cholesterol, altering immunoregulatory sterols, and producing novel secondary metabolites^35^.

We also investigated proteins classified into the “transporter activity” term (GO:0005215), based on GO analysis by “Molecular Function”. Three and 15 “transporter activity” proteins, respectively, were over- and under-represented in the Beijing B0/W148 strains. Five of the under-represented proteins are classified as ABC transporters and, of these, PstB and PstS1 belong to a single operon involved in phosphate import during fasting, which is a characteristic of bacteria inside phagosomes^34^. In addition, PstS1 is overexpressed during phosphate starvation^36^.

Another interesting transport protein is CtpF, which was not detectable in the Beijing B0/W148 strains. This protein is a P-type ATPase and potential alkali/alkaline earth metal cation transporter. CtpF is the only P-type ATPase gene that is regulated by the global latency regulator, DosR, and is highly overexpressed under conditions of hypoxia. From the genomic data we determined that the corresponding gene had a single nucleotide deletion (Rv1997: 2241032 delG) leading to a frame shift mutation. This mutation is not specific to Beijing B0/W148 strains, but is rather a general characteristic of the Beijing family.

It should be noted that GO analysis reflects the independent roles of differentially abundant proteins, and does not take into account potentially related changes in the bacterial cell. To estimate the effects of co-regulated proteins, we used a gene regulatory network for MBT from a recently published study by Peterson *et al*.^24^. This analysis revealed that there was a decreased representation of 11 proteins from the DosR system in Beijing B0/W148 strains. The MTB DosR system has been well documented; in H37Rv, it is induced under three conditions that inhibit aerobic respiration: hypoxia, NO, or CO^26,37–39^. It is well known that, during the exponential phase of MTB growth, the levels of DosR transcripts are constitutively higher in Beijing B0/W148 strains than in H37Rv^40^,^41^. In contrast, Badillo-López *et al*. demonstrated that, during hypoxia, the level of DosR is significantly lower in the Beijing strains than in H37Rv^42^. This is explained by the fact that Beijing strains of MTB implement an alternative response to hypoxic stress than that used by H37Rv^42^. Our results demonstrated an under-representation of DosR-regulated proteins in the Beijing B0/W148 strains during the stationary growth phase. This might be due to pre-adaptation of bacterial cells to the potentially low levels of oxygen under prolonged cultivation *in vitro.*

Of interest, the transcription factor Rv0081 of the DosR regulon was not detectable in any Beijing B0/W148 strain. This protein was recently described by Galagan *et al*. as a regulator hub during the response to hypoxia^26^ and, together with Rv1049, controls the mce1 operon, consisting of six *mce* genes (Rv0169–Rv0174), two *yrbE* genes (Rv0167, Rv0168), and four *mce*-associated genes (Rv0175–Rv0178). In our study we found that four Mce proteins (Rv0169, Rv0170, Rv0172, and Rv0174), and one Mce-associated protein (Rv0176) were under-represented in the Beijing B0/W148 strains. The proteins encoded by the *mce*1 operon are thought to be ABC transporters involved in the transport of phospholipids, and are required for mycobacterial survival in macrophages or mouse models of infection^36^,^43^, and deletion of the *mce*1 operon results in a hypervirulent phenotype^36^. Notably, these proteins were not defined in the GO analysis, highlighting the need to use different approaches to functionally assess proteins and better understand how they work in concert to ensure cell survival.

In earlier study, the efflux pump proteins Rv0341, Rv2688c, Rv3728, are found exclusively in Beijing MTB strains^19^. In contrast, we found that Rv0341 was present in all strains tested, including H37Rv, and that its abundance was similar between Beijing B0/W148 strains and H37Rv. However, Rv2688 and Rv3728 proteins were not identified in our dataset. Notably, we observed that the Rv3728 gene carried a B0/W148-specific mutation, leading to the formation of an early stop codon, indicating why this protein may be absent from the B0/W148 proteome.

Beijing B0/W148 strains belong to the “modern” Beijing sublineage^6^, which differ from the “ancient” sublineage by the over-representation of the proteins Rv0450c and Rv3137, and the under-representation of Rv1269c and Rv3283^19^. Also in our data, the abundance of Rv3283 (SseA) was dramatically lower in Beijing B0/W148 strains than in H37Rv (fold change = 0.04). This protein is a predicted thiol-oxidoreductase and, together with the superoxide detoxifying enzyme, SodA (Rv3846) and an integral membrane protein, DoxX (Rv3005c), it forms a membrane-associated oxidoreductase complex (MRC). Loss of any MRC component is correlated with defective recycling of mycothiol, which is a functional analog of glutathione in MTB^44^. The decreased abundance of Rv3283 (SseA) in «modern» Beijing cells might lead to the accumulation of oxidative cellular damage, caused by reactive oxygen species (ROS).

Taken together, our data suggest that the distinctive proteomic features of Beijing B0/W148 strains are likely to contribute to their enhanced virulence and successful geographical spread. We observed an increased abundance of enzymes responsible for long-chain fatty acid biosynthesis, which coincided with a decrease in proteins responsible for the degradation of these molecules. Mycobacteria can use long chain fatty acids (up to 86–95 carbon atoms in length) to produce mycolic acids and various lipids that are considered to be the major virulence factors of MTB, in particular during the early stage of infection, when bacilli encounter host macrophages. We also observed an increased abundance of the HsaA protein involved in steroid degradation. In the intracellular environment, MTB uses cholesterol as an energy source and for the biosynthesis of the cell wall lipids. The difference we observed in the abundance of HsaA protein could increase the survival of MTB in host macrophages, a known characteristic of Beijing B0/W148 strains^7^,^45^. In addition, we observed a decreased abundance of proteins encoded by *mce*1 operon genes, the deletion of which is known to lead to a hypervirulent phenotype^36^. Our data also provide a possible basis for the well-known ease with which stains with the Beijing B0/W148 genotype develop drug resistance. We confirmed very low levels of SseA protein in B0/W148 strains, which is likely to lead to an accumulation of ROS, followed by DNA damage. The latter has the potential to generate a wide range of genetic variants, supporting the survival of MTB populations under positive selection, in particular during drug therapy.

## Materials and Methods

### Mycobacteria cultivation conditions

Eight strains of MTB were used; seven Beijing B0/W148 cluster strains, which were treated as an experimental group, and the control H37Rv strain (Table 1). *Mycobacterium tuberculosis* strains were grown in Middlebrook 7H11 media with OADC supplement at 35 °C without shaking for 14–16 days to a cell density of 1 ± 2 × 10^8^ cells ml^−1^. Each strain was grown in three biological replicates. The bacterial cells were washed in Tris-HCl, PBS+2%, Triton-X100 (pH 7.5–8) and incubated at 80 °C for 20 min. Further cells pellet was received by centrifugation at 4,500 g, 4 °С for 15 min and stored at −80 °C until required.

### Whole genome sequencing and PCR analysis of Beijing B0/W148 strains

DNA extraction was performed as previously described^46^. Strains of the Beijing B0/W148-cluster were detected using a PCR assay^47^. Large chromosomal inversions in the B0/W148 genome were detected as described previously^9^. Spoligotyping and 24-locus VNTR typing were performed as previously described^48^,^49^, as were genome sequencing and SNP calling^9^.

### Protein Extraction from *M. tuberculosis*

Bacterial cell pellets were resuspended in 50 μL lysozyme and 100 μL 100 mМ TrisHСl pH 7.6, with 3 μL of Protease inhibitor Mix (GE Healthcare, USA). Cells were disrupted using a bead-beating homogenizer (MPBio, FastPrep-24, USA) with 0.5 mm silica-zirconium beads for 4 min, followed by 5 min on ice. For protein solubilization, SDS (Panreac, Spain) was added to the collected suspension to a final concentration of 10%. To reduce disulfide bonds, DTT (BioRad, USA) was added to a final concentration of 100 mM. Samples were then incubated at 60 °C for 30 min, centrifuged at 13,000 g at 4 °С for 5 min, and the supernatant used as protein solution. Protein concentration was measured by the Bradford method using the Bradford Protein Assay Kit (Bio Rad, USA).

### Trypsin Digestion

Protein samples (200 μg) were loaded onto a 7.5% SDS-PAGE gel and separated by electrophoresis at 20 mA for 20 min and 40 mA overnight using a PROTEAN II system (Bio-Rad, USA). The gel was stained using a Colloidal Blue Staining Kit (Invitrogen, USA)^50^. Proteolytic in gel digestion was performed with trypsin (Trypsin Gold, Mass Spectrometry Grade, Promega, USA) as described previously^51^. Cleavage was stopped by adding 5% formic acid (FA) and peptides were extracted in a solution containing 50% ACN and 5% FA (2v/v), followed by extraction in 75% ACN and 5% FA (2v/v). Peptides were concentrated by Speedvac and dissolved in 20 μL 1% acetic acid. Supernatant peptides were removed and cleaned using C18 Sep-Pak columns (Waters, USA).

### LC-MS/MS analysis

Analysis was performed on a TripleTOF 5600+ mass-spectrometer with a NanoSpray III ion source (AB Sciex, Canada) coupled to a NanoLC Ultra 2D+ nano-HPLC system (Eksigent, Singapore). The HPLC system was configured in a trap-elute mode. For a sample loading buffer and buffer A, a mix of 98.9% water, 1% methanol, and 0.1% formic acid (v/v) was used. Buffer B was 99.9% acetonitrile and 0.1% formic acid (v/v). Samples were loaded on a trap column Chrom XP C18, 3 mm, 120 Å, 350 mm × 0.5 mm (Eksigent, Singapore) at a flow rate of 3.5 μl/min over 10 min and eluted through the separation column 3C18-CL-120 (3 mm, 120 Å) 75 mm × 150 mm (Eksigent, Singapore) at a flow rate of 300 nl/min. The gradient was from 5 to 40% of buffer B in 120 min. The column and the pre-column were regenerated between runs by washing with 95% buffer B for 7 min and equilibrated with 5% buffer B for 25 min. Between samples, to ensure the absence of carryover, both the column and the precolumn were thoroughly washed with blank trap-elute gradient, including 5–7 min of 5-95-95-5% waves of buffer B followed by 25 min of equilibration with 5% buffer B.

Mass spectra were acquired in the positive ion mode. Information-dependent mass-spectrometer experiments included one survey MS1 scan followed by 50 dependent MS2 scans. MS1 acquisition parameters were as follows: mass range for analysis and subsequent ion selection for MS2 analysis was 300–1250 m/z, signal accumulation time was 250 ms. Ions for MS2 analysis were selected on the basis of intensity, with a threshold of 200 cps and charge state between 2 and 5. MS2 acquisition parameters were as follows: resolution of quadrupole was set to UNIT (0.7 Da), measurement mass range was 200–1800 m/z, optimization of ion beam focus was set to obtain maximal sensitivity, and signal accumulation time was 50 ms for each parent ion. Collision activated dissociation was performed with nitrogen gas, with collision energy ramping from 25 to 55 V within the 50 ms signal accumulation time. Analyzed parent ions were sent to a dynamic exclusion list for 15 sec, in order to collect the next MS2 spectra of the same compound around its chromatographic peak apex (the minimum peak width throughout the gradient was approximately 30 s).

### Protein identification

Raw data (.wiff files) were converted to Mascot Generic Format (.mgf files, peak lists) using the command-line program, AB SCIEX MS Data Converter v.1.3 (AB SCIEX, Framingham, MA, USA) and the “-proteinpilot” parameter. Mascot v. 2.2.07 was used for identification against the *Mycobacterium tuberculosis* H37Rv sequence database (3,932 amino acid sequences, including 26 contaminant sequences) downloaded from the RefSeq database^52^ (RefSeq: NC_000962.3). In the Mascot search results, when the significance threshold was set at 0.05, the individual ions score was >11 (Table S7). The peptide false discovery rate (peptide FDR) was calculated using Decoy database analysis. Frequently observed contaminants, such as trypsin, bovine proteins and human keratins, were removed from the results, along with proteins supported by a single unique peptide. The mass spectrometry proteomics data have been deposited to the ProteomeXchange Consortium^53^ via the PRIDE partner repository with the dataset identifier PXD002542 (Reviewer account details: Username - reviewer44310@ebi.ac.uk and password - KJzyqV1z).

Additionally, for protein identification, .wiff data files were analyzed with ProteinPilot^TM^ (AB Sciex, Canada) software version 4.5, revision 1656, using search algorithm Paragon 4.5.0.0, revision 1654 (AB Sciex, Canada) and a standard set of identification settings to search against the RefSeq database (RefSeq: NC_000962.3), supplemented with sequences of trypsin and common protein contaminants, to give a total of 4298 protein sequences. Peptide identifications were processed with default settings by using the ProGroup algorithm integral to ProteinPilot software. The software algorithm includes any modification listed in UniMod, based on the estimated probability of its occurrence^54^. The final protein identification list for each sample was obtained by leaving out protein identifications with unused scores below the threshold calculated by the ProteomicS Performance Evaluation Pipeline (PSPEP) algorithm for 1% global FDR from fit (which is defined using protein hits for decoy reversed sequences in the provided database)^55^. In addition, only identifications for which two or more unique peptides with confidence scores above the threshold calculated by PSPEP software for 1% global FDR were retained.

We used TubercuList version 2.6 (http://tuberculist.epfl.ch/) and PSORTdb v 3.0 (http://db.psort.org/) databases to determine functional categories and localization of the identified proteins.

### Label-free protein quantitation

For label-free quantitation, raw MS data files (.wiff files) were imported and processed in Progenesis LC-MS software v.4.1 (Nonlinear Dynamics, Newcastle, UK). The sample of *M. tuberculosis* H37Rv with the highest number of MS/MS spectra was set as the reference and all other runs were aligned to it. Searches were performed using Mascot Search Engine as described in “Protein identification” section. The results of peptide quantitation were normalized using an iterative median-based normalization as implemented in the Progenesis software. Differences in the abundance of a protein between the three biological replicates of *M. tuberculosis* H37Rv and all Beijing B0/W148 cluster strains were evaluated using a two-sided unpaired Student’s T-test. P-values < 0.05 were considered statistically significant. Adjusted p-values for multiple tests (q-values) were generated using the Benjamini–Hochberg method^56^.

### Gene ontology analysis

To functionally characterize differentially abundant proteins for biological interpretation, Gene Ontology (GO) analysis was performed. Gene Ontology annotation for H37Rv proteins was obtained from UniProt^57^ using the ID mapping function (http://www.uniprot.org/uploadlists/). The TopGO R package from Bioconductor was used for GO enrichment analysis^58^. A two-tailed Fisher’s Exact Test was used to measure the significance of enrichment. Proteins assigned to enriched GO categories (p-value < 0.05) were grouped according to the PANTHER classification system^59^.

## Additional Information

**How to cite this article**: Bespyatykh, J. *et al*. Proteome analysis of the *Mycobacterium tuberculosis* Beijing B0/W148 cluster. *Sci. Rep.* **6**, 28985; doi: 10.1038/srep28985 (2016).

## Supplementary Material

Supplementary Information

Supplementary Information

Supplementary Information

Supplementary Information

Supplementary Information

Supplementary Information

Supplementary Information

## Figures and Tables

**Table 1:** Description of *M. tuberculosis* strains.

Sample	Сlade	SIT №	24-VNTR*	Ac Numb
H37Rv	H37Rv	SIT-451	233′226133321242534233552	NC_000962.3
Sp1**	Beijing	SIT-1	223325173533424672454433	SRX216883
Sp7**	Beijing	SIT-1	223325173533424672454433	SRX216889
Sp10	Beijing	SIT-1	223325173533424672444433	SRX216892
Sp13	Beijing	SIT-1	223325173533424672454433	SRX216895
Sp22	Beijing	SIT-1	223325173533424572454433	SRX216900
Sp27	Beijing	SIT-1	223325173533424572454433	
Sp45	Beijing	SIT-1	223325173533424672454433	

^*^24 – VNTR: s154, s580, s960, s1644, s2059, s2531, s2687, s2996, s3007, s3192, s4348, s802, s2165, s2461, s577, s2163, s4052, s4156, s424, s1955, s2347, s2401, s3171, s3690^48^.

^**^Published in ref. 9.

**Table 2:** Enriched functional clusters of differential proteins discussed in the text.

Gene	Synonym	Reg	Functional_category	Product	log2 fold	p-value
transporter activity (GO:0005215)						
fadD15	Rv2187	over	Lipid metabolism	long-chain-fatty-acid–CoA ligase FadD15	0.64	0.049358831
–	Rv2971	over	Intermediary metabolism and respiration	oxidoreductase	0.5	0.041065417
–	Rv0073	under	cell wall and cell processes	glutamine ABC transporter ATP-binding protein		
–	Rv0143c	under	cell wall and cell processes	transmembrane protein		
pstB	Rv0933	under	cell wall and cell processes	phosphate ABC transporter ATP-binding protein PstB	−2.41	4.65402E-07
pstS1	Rv0934	under	cell wall and cell processes	phosphate ABC transporter substrate-binding lipoprotein PstS	−1.12	0.00155862
oppD	Rv1281c	under	cell wall and cell processes	oligopeptide ABC transporter ATP-binding protein OppD	−0.6	0.045017563
atpA	Rv1308	under	Intermediary metabolism and respiration	ATP synthase subunit alpha	−0.46	0.001330189
atpD	Rv1310	under	Intermediary metabolism and respiration	ATP synthase subunit beta	−0.66	0.00060944
glnQ	Rv2564	under	cell wall and cell processes	glutamine ABC transporter ATP-binding protein	−0.76	0.00036177
–	Rv2690c	under	cell wall and cell processes	integral membrane protein		
lipid metabolic process (GO:0006629)						
fadD15	Rv2187	over	Lipid metabolism	long-chain-fatty-acid–CoA ligase FadD15	0.64	0.049358831
–	Rv2277c	over	Intermediary metabolism and respiration	glycerolphosphodiesterase		
agpS	Rv3107c	over	Lipid metabolism	alkyldihydroxyacetonephosphate synthase	3.5	0.022585521
hsaA	Rv3570c	over	Intermediary metabolism and respiration	flavin-dependent monooxygenase oxygenase subunit HsaA	1	0.043815093
rmlA	Rv0334	under	Intermediary metabolism and respiration	glucose-1-phosphate thymidylyltransferase	−1.41	7.01184E-05
pks6	Rv0405	under	Lipid metabolism	membrane bound polyketide synthase		
fadB2	Rv0468	under	Lipid metabolism	3-hydroxybutyryl-CoA dehydrogenase	−1.33	2.15157E-07
fadB	Rv0860	under	Lipid metabolism	fatty oxidation protein FadB	−0.47	0.008655458
echA6	Rv0905	under	Lipid metabolism	enoyl-CoA hydratase EchA6	−0.96	0.002614821
fadD21	Rv1185c	under	Lipid metabolism	fatty-acid–CoA ligase FadD21	−1.35	0.004873026
–	Rv3230c	under	Intermediary metabolism and respiration	stearoyl-CoA 9-desaturase electron transfer partner	−1.38	0.03233442
dprE2	Rv3791	under	Lipid metabolism	decaprenylphosphoryl-D-2-keto erythropentose reductase	−0.85	0.003881878
pks2	Rv3825c	under	Lipid metabolism	phthioceranic/hydroxyphthioceranic acid synthase	−1	0.03160682

**Table 3:** Differential proteins identified on the gene regulatory network.

Synonym	Gene	Reg	TF	Product	Functional_category	log2 fold	p-value
Rv0509	–	over	Rv1353c	glutamyl-tRNA reductase	Intermediary metabolism and respiration		
Rv1464	–	over	Rv1460	cysteine desulfurase	Intermediary metabolism and respiration	1.56	0.0489072
Rv3094c	fadE2	over	Rv0494; Rv3095	hypothetical protein	Intermediary metabolism and respiration	9.61	0.0386941
Rv3494c	yrbE1A	over	Rv0022c	Mce family protein Mce4	virulence, detoxification, adaptation	1.46	0.0259927
Rv3570c	mce1A	over	Rv0678; Rv1353c; Rv3574	flavin-dependent monooxygenase oxygenase subunit HsaA	Intermediary metabolism and respiration	1	0.0438151
Rv0034	–	under	Rv3249c	hypothetical protein	Intermediary metabolism and respiration		
Rv0035	–	under	Rv3249c	fatty-acid–CoA ligase FadD34	Lipid metabolism		
Rv0081	bioF2	under	Rv0081	HTH-type transcriptional regulator	Regulatory proteins		
Rv0087	acpA	under	Rv0081	formate hydrogenase HycE	Intermediary metabolism and respiration		
Rv0101	–	under	Rv0047c; Rv2069; Rv0324	peptide synthetase Nrp	Lipid metabolism		
Rv0154c	fadD34	under	Rv1423	acyl-CoA dehydrogenase FadE2	Lipid metabolism	−0.59	0.0008057
Rv0169	–	under	Rv0023; Rv0081; Rv0757; Rv1049; Rv3416	Mce family protein Mce1A	virulence, detoxification, adaptation	−1.44	5.763E-06
Rv0170	–	under	Rv0023; Rv0081; Rv1049; Rv3416	Mce family protein Mce1B	virulence, detoxification, adaptation	−1.38	0.0017153
Rv0172	sdaA	under	Rv0023; Rv0081; Rv1049	Mce family protein Mce1D	virulence, detoxification, adaptation	−1.38	0.0001941
Rv0173	glyA2	under	Rv1049	Mce family lipoprotein LprK	cell wall and cell processes	−1.29	7.012E-05
Rv0174	-	under	Rv0023; Rv0081; Rv1049	Mce family protein Mce1F	virulence, detoxification, adaptation	−0.84	7.012E-05
Rv0176	–	under	Rv1049	Mce associated transmembrane protein	cell wall and cell processes	−1.81	0.0175553
Rv0241c	–	under	Rv0238; Rv1049	3-hydroxyacyl-thioester dehydratase HtdX	Intermediary metabolism and respiration	−0.86	0.0386941
Rv0243	–	under	Rv0238	acetyl-CoA acetyltransferase FadA	Lipid metabolism	−0.91	0.0166606
Rv0675	–	under	Rv0674	enoyl-CoA hydratase EchA5	Lipid metabolism	−1.1	0.0007597
Rv0768	–	under	Rv0576; Rv1255c	aldehyde dehydrogenase AldA	Intermediary metabolism and respiration		
Rv0824c	hycD	under	Rv0472c	acyl-ACP desaturase DesA	Lipid metabolism	−1.98	5.763E-06
Rv0989c	hycP	under	Rv0767c	polyprenyl-diphosphate synthase GrcC	Intermediary metabolism and respiration		
Rv1094	hycQ	under	Rv0472c	acyl-ACP desaturase DesA	Lipid metabolism	−1.91	5.196E-09
Rv1192	hycE	under	Rv2034; Rv0081	hypothetical protein	cell wall and cell processes	−1.91	0.0006327
Rv1386	-	under	Rv1033c; Rv2359	PE family protein PE15	PE/PPE		
Rv1856c	fcoT	under	Rv1353c	oxidoreductase	Intermediary metabolism and respiration	−1.06	0.037951
Rv1997	ctpF	under	Rv3133c	cation transporter ATPase F	cell wall and cell processes		
Rv2004c	–	under	Rv3133c	hypothetical protein	Regulatory proteins	−1.45	0.0004367
Rv2005c	nrp	under	Rv3133c	universal stress protein	virulence, detoxification, adaptation	−1.24	0.023446
Rv2029c	gmhB	under	Rv3133c	6-phosphofructokinase PfkB	Intermediary metabolism and respiration	−3	0.0006327
Rv2048c	hddA	under	Rv0767c	polyketide synthase	Lipid metabolism	−1.38	0.0393413
Rv2103c	msrA	under	Rv1990c	ribonuclease VapC37	virulence, detoxification, adaptation		
Rv2410c	–	under	Rv0022c	hypothetical protein	Intermediary metabolism and respiration	−1.42	0.0082996
Rv2623	–	under	Rv3133c	universal stress protein	virulence, detoxification, adaptation	−1.26	0.0157466
Rv2626c	ptbB	under	Rv3133c	hypoxic response protein	Regulatory proteins	−1.56	0.0216908
Rv3130c	pntAa	under	Rv3133c	diacyglycerol O-acyltransferase	Lipid metabolism	−2.29	0.0001161
Rv3131	pntAb	under	Rv3133c	NAD(P)H nitroreductase	Intermediary metabolism and respiration	−1.69	0.0026414
Rv3400	pntB	under	Rv0135c	hydrolase	Intermediary metabolism and respiration	−0.93	0.0451627
Rv3509c	yrbE1B	under	Rv0324	acetohydroxyacid synthase large subunit	Intermediary metabolism and respiration	−0.66	0.0080181
Rv3602c	mce1B	under	Rv1353c	pantothenate synthetase	Intermediary metabolism and respiration	−1.25	0.0014439
Rv3825c	mce1C	under	Rv0757	phthioceranic/hydroxyphthioceranic acid synthase	Lipid metabolism	−1	0.0316068
