# Supplementary material for: Proteome analysis of the Mycobacterium tuberculosis Beijing B0/W148 cluster
Source: Sci Rep. 2016 Jun 30;6:28985. doi: 10.1038/srep28985 (PMC4928086; doi:10.1038/srep28985)
Supplement: Supplementary Information [file srep28985-s1.pdf]

Proteome analysis of the *Mycobacterium tuberculosis* Beijing B0/W148 cluster

Julia Bespyatykh, Egor Shitikov, Ivan Butenko, Ilya Altukhov[1], Dmitry Alexeev, Igor Mokrousov, Marine Dogonadze, Viacheslav Zhuravlev, Peter Yablonsky, Elena Ilina and Vadim Govorun

Figure S1: A volcano plot representation of the differentially expressed genes in a pair-wise comparison of Beijing B0/W148 strains and H37Rv. The significance cut-off was set to a p-value of 0.05 (-$\log_{10}$ (adjusted p-value) > 1.3), the biological cut-off was set to a fold change of 2 (-1 >= $\log_2$ (fold change) >= 1). The five colors represent insignificant genes (grey), statistically and biologically over-represented genes (red), statistically and biologically under-represented genes (green), statistically but no biologically over-represented genes (orange) and statistically but not biologically under-represented genes (light-green). DosR regulon proteins are highlighted by black circles. X-axis and Y-axis limits were set to (-5…5) and (0…7) respectively.

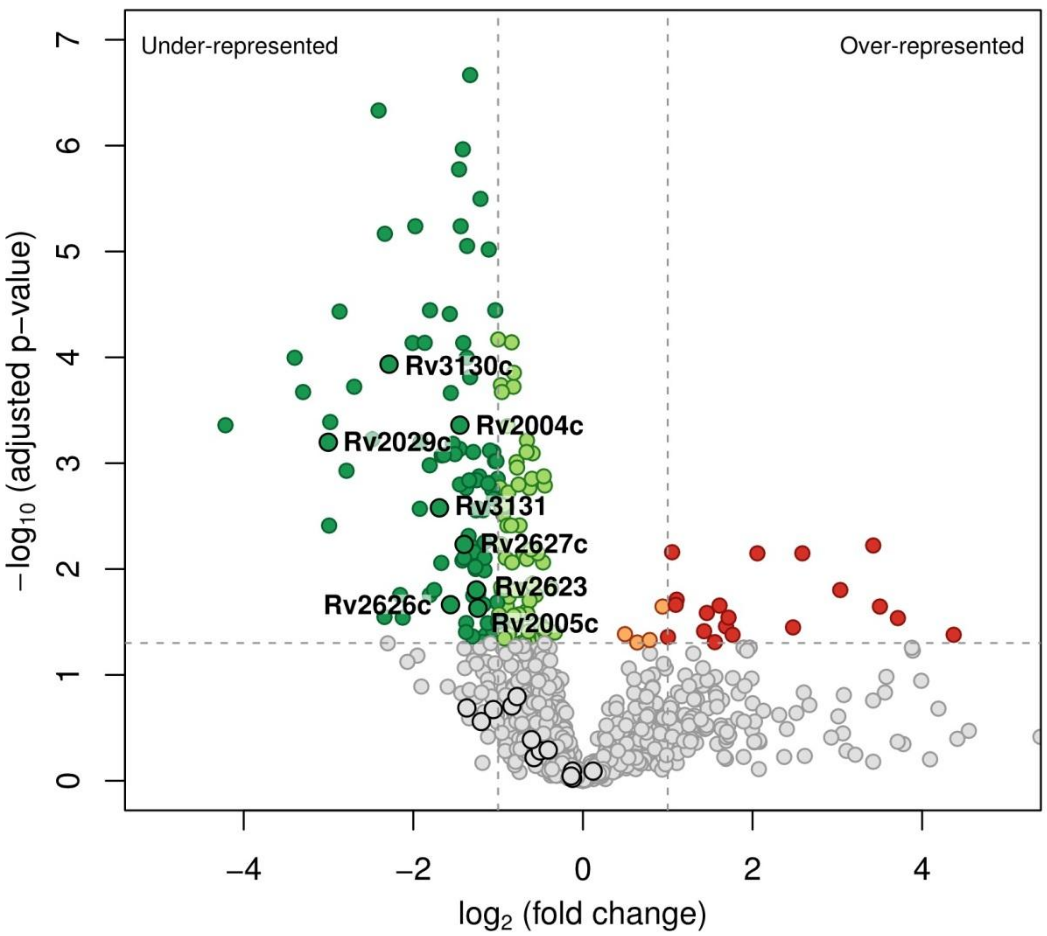

Figure S2: Representative enriched functional clusters for differential proteins. Black color indicates under-represented proteins in Beijing B0/W148 compared to H37Rv strains. Gray color indicates over-represented proteins. A) Distribution of proteins in the main categories. B) Detail enriched of proteins in «biological process» category.

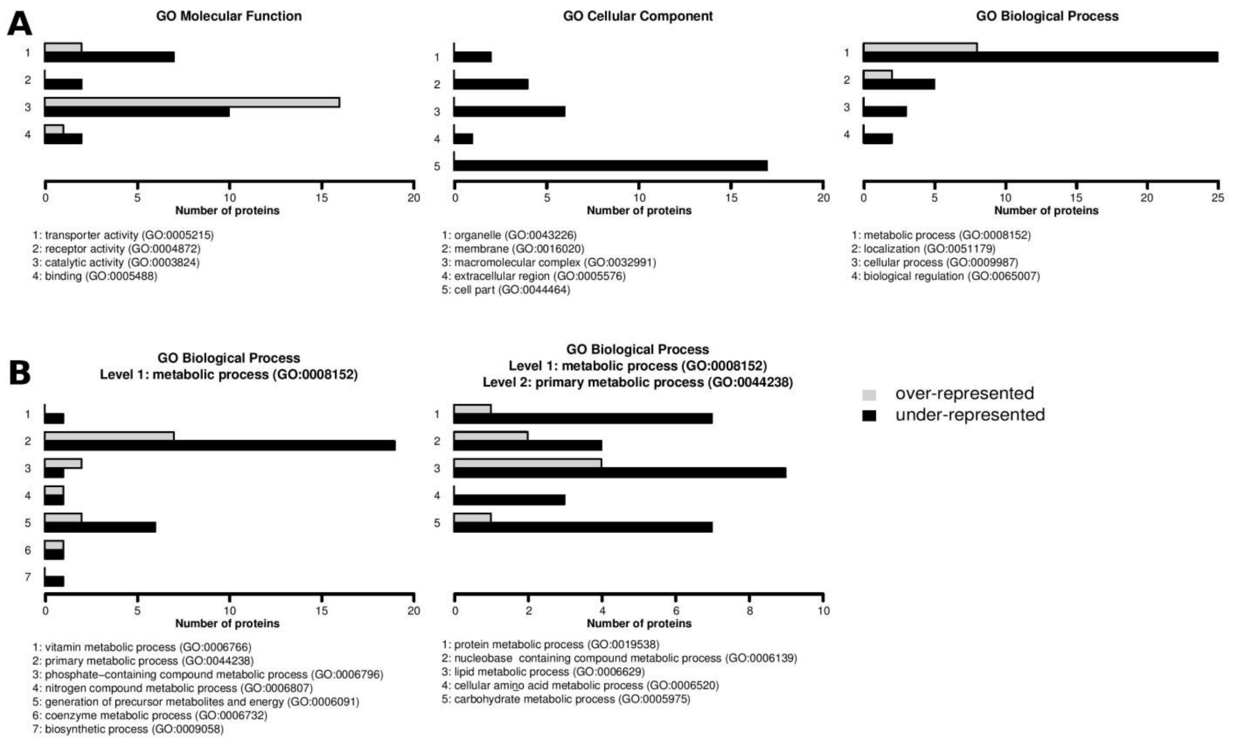

Table S7. Results of Mascot identification

Strain	Sample	Rep	Number of identified spectra	Number of identified unique peptides	Total number of spectra	Individual ion score cutoff (p < 0.05)	FDR
Sp1	B063	1	25367	9389	56707	11	0.02
Sp1	B066	2	21869	7924	47233	11	0.01
Sp1	B069	3	20006	7644	46718	11	0.01
Sp7	B072	1	19040	7724	43103	11	0.01
Sp7	B078	2	20170	7555	45216	11	0.01
Sp7	B091	3	7205	3987	45627	11	0.01
Sp10	B027	1	18046	8284	48558	11	0.01
Sp10	B030	2	15856	7362	46669	11	0.01
Sp10	B033	3	12476	6605	42132	11	0.01
Sp13	B045	1	14227	6804	44543	11	0.01
Sp13	B048	2	15885	7114	46620	11	0.01
Sp13	B051	3	10632	5536	42055	11	0.02
Sp22	B054	1	10568	5649	38799	12	0.01
Sp22	B057	2	15182	5892	42308	11	0.01
Sp22	B060	3	13934	6154	39110	11	0.01
Sp27	B013	1	15422	7283	43560	11	0.01
Sp27	B016	2	17734	8348	44289	11	0.01
Sp27	B017	3	10402	5422	45036	11	0.02
Sp45	B020	1	17321	7730	47792	11	0.01
Sp45	B023	2	16391	8239	45221	11	0.01

Sp45	B024	3	14141	6767	45474	11	0.01
H37Rv	B092	1	12377	6354	51431	11	0.01
H37Rv	B093	2	12997	6379	52406	11	0.01
H37Rv	B094	3	9373	4709	48387	11	0.01
